# Feasibility of Using a Novel 2.45 GHz Double Short Distance Slot Coaxial Antenna for Minimally Invasive Cancer Breast Microwave Ablation Therapy: Computational Model, Phantom, and *In Vivo* Swine Experimentation

**DOI:** 10.1155/2018/5806753

**Published:** 2018-05-08

**Authors:** R. Ortega-Palacios, C. J. Trujillo-Romero, M. F. J. Cepeda Rubio, A. Vera, L. Leija, Jose L. Reyes, M. C. Ramírez-Estudillo, F. Morales-Alvarez, M. A. Vega-López

**Affiliations:** ^1^Electrical Engineering Department, Bioelectronics Section, LAREMUS Laboratory, CINVESTAV, Mexico City, Mexico; ^2^Division of Medical Engineering Research, National Institute of Rehabilitation, Mexico City, Mexico; ^3^División de Estudios de Posgrado e Investigación, Tecnológico Nacional de México, Instituto Tecnológico de la Laguna, Torreón, Mexico; ^4^Department Physiology, Biophysics and Neurosciences, Center for Research and Advanced Studies, National Polytechnic Institute, Mexico City, Mexico; ^5^Departamento de Infectómica y Patogénesis Molecular, Laboratorio de Inmunobiología de las Mucosas, CINVESTAV, Mexico City, Mexico; ^6^CENID Microbiología Animal, INIFAP-SAGARPA, Mexico City, Mexico

## Abstract

Microwave ablation (MWA) by using coaxial antennas is a promising alternative for breast cancer treatment. A double short distance slot coaxial antenna as a newly optimized applicator for minimally invasive treatment of breast cancer is proposed. To validate and to analyze the feasibility of using this method in clinical treatment, a computational model, phantom, and breast swine *in vivo* experimentation were carried out, by using four microwave powers (50 W, 30 W, 20 W, and 10 W). The finite element method (FEM) was used to develop the computational model. Phantom experimentation was carried out in breast phantom. The *in vivo* experimentation was carried out in a 90 kg swine sow. Tissue damage was estimated by comparing control and treated micrographs of the porcine mammary gland samples. The coaxial slot antenna was inserted in swine breast glands by using image-guided ultrasound. In all cases, modeling, *in vivo* and phantom experimentation, and ablation temperatures (above 60°C) were reached. The *in vivo* experiments suggest that this new MWA applicator could be successfully used to eliminate precise and small areas of tissue (around 20–30 mm^2^). By modulating the power and time applied, it may be possible to increase/decrease the ablation area.

## 1. Introduction

Tumor ablative therapies are either chemical or thermal treatment applied to a tumor tissue in order to get partial or total tumor destruction. The advantages of ablative therapies include faster recovery, lower cost, and a less invasive procedure; moreover, these therapies can be performed in an ambulatory surgery setting under local anesthesia. Another advantage of ablative therapy is the cosmetic breast preservation, because this treatment is less aggressive and allows keeping the shape of the breast [1, 2]. Many thermal ablation modalities have been researched, including cryoablation, laser ablation, high-intensity focused ultrasound, radiofrequency ablation, and microwave ablation [3–5]. Microwave ablation (MWA) not only is an alternative for breast cancer therapy but also can potentiate the effects of chemotherapy [6]. Electromagnetic microwave radiation excites water molecules in the tissue; this produces friction and heat, which induce cellular death [7]. The microwave power produces local heat in the tumor without damaging the surrounding healthy tissue due to the difference in electric properties between healthy and tumor breast tissues.

Microwave ablation has several theoretical advantages over other therapies [8, 9], which would make it more attractive to treat breast tumors. Some benefits of MWA are higher tumor temperatures, less time to get ablation, and the possibility to use multiple probes to treat several tumors at the same time [10–12]. These benefits have motivated the researchers to improve the original antennas to get more effective tumor treatment. The studies have been focused on coaxial thin interstitial antennas [13–15], which could be classified as monopole, dipole, or slot antennas [16]. It is important to remark that, to our best knowledge, there are few reports in the literature concerning MWA breast tumor treatment [17–21].

During clinical MWA treatment, it is necessary to control tissue temperature and monitor the behavior of the surrounded healthy tissue. Therefore, the treatment models help us to explain the ablation process. The finite element method (FEM) is a numerical technique used to predict the antenna behavior over the treated tissue. Computational model results are essential because they allow us to make predictions about what will occur in real ablation treatment.

One of the parameters that we took into account for the design of the applicator was the standing wave ratio (SWR), which determines the energy transfer to the radiated media. We have had good SWR results (near to 1.0) when using the double short distance slot antenna, which allows better incident power distributions in the tissue, reduces the reflected power along the applicator, and avoids damage to healthy tissue around the incision [22].

The main goal of this work is to validate the double short distance slot coaxial antenna. A computational model, phantom experimentation, and breast swine *in vivo* experimentation were carried out. To evaluate the antenna, a breast phantom at 2.45 GHz was developed. For the *in vivo* experimentation, a 90 kg swine sow was used. The evaluations (phantom and *in vivo*) were carried out by using four different microwave powers (10–50 W) and exposition times.

## 2. Materials and Methods

### 2.1. Antenna Design

**Figure 1:**
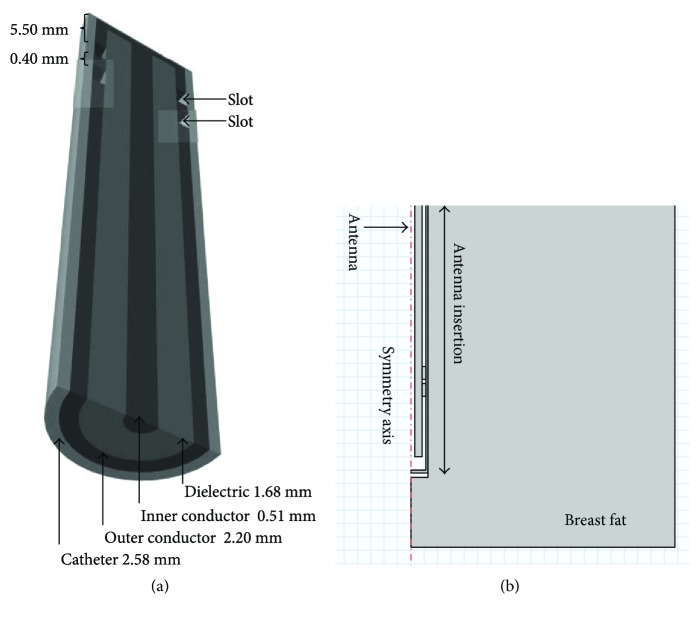
Double short distance slot antenna. (a) Geometry of the double short distance coaxial slot antenna designed to treat breast cancer tissue at 2.45 GHz. (b) 2D axisymmetric geometry used for the FEM models.

Due to the easiness of construction, the coaxial slot antenna (applicator) was chosen; moreover, its diameter (approximately 2.5 mm) allows minimally invasive treatments. The geometry parameters of the antenna and the slot spacing were designed according to the effective wavelength in breast cancer tissue at 2.45 GHz, which was calculated by using

(1) $$\begin{array}{c} \lambda_{eff}=\frac{c}{f\sqrt{\varepsilon_{r}}}, \end{array}$$

where the speed of light in free space is *c* (m/s), the operating frequency of the microwave generator is *f* (2.45 GHz), and the relative permittivity of the breast fat tissue is *ε*_r_ = 5.15 (mean value). The slot distance was calculated based on the effective wavelength in breast cancer tissue at 2.45 GHz by using (1). The slot spacing length corresponds to 0.025 *λ*_eff_ = 0.40 mm.  Figure 1(a) illustrates the double short distance coaxial antenna design. The antenna was built in a semirigid coaxial cable (UT-085). This cable has a center conductor diameter of 0.51 mm, an outer conductor diameter of 2.20 mm, and a dielectric diameter of 1.68 mm. The antenna was encased in a polytetrafluoroethylene (PTFE) catheter (2.58 mm) to avoid adhesion of the antenna to ablated tissue [18]. The double short distance slot antenna was modeled and optimized. The antenna reported here was chosen because the obtained SWR allows a better power transmission to the tissue; that is, a better energy distribution in the tissue was achieved. Moreover, this antenna reduces the reflected power along the applicator avoiding damage to healthy tissue around the incision.

### 2.2. Computational Model

The finite element method (FEM) was used to simulate the performance of the microwave coaxial slot antenna.  Figure 1(b) presents a 2D axisymmetric geometry model. The coaxial antenna was modeled to analyze the heat transfer in breast tissue under the same conditions of *in vivo* experimentation.

Maxwell's equations were used to model the ablation process. To develop a proper model, it was necessary to establish tissue electromagnetic properties (permittivity and conductivity) and initial boundary conditions. Equation (2) represents the external heat source related to the electromagnetic field:

(2) Qext=12Reσ−jωεE·E∗,

where *σ* and *ε* are tissue conductivity and permeability, respectively, and *ω* is the frequency of the source and *E* is the electric field generated by the proposed antenna (Re is the real part). Equation (2) is coupled to the Pennes Bioheat equation (3), which was used in order to model microwave thermal effects in breast tissue. Equation (3) describes the problem of stationary heat transfer as

(3) $$\begin{array}{c} \nabla \cdot \left(-k\nabla T\right)=\rho_{b}C_{b}\omega_{b}\left(T_{b}-T\right)+Q_{met}+Q_{\text{ext}}, \end{array}$$

where tissue thermal conductivity is *k* (W/(m·K)), the blood density is ρ_b_ (kg/m^3^), the blood specific heat capacity is *C*_b_ (J/(kg·K)), the blood perfusion rate is *ω*_b_ (mL/min/kg), the metabolism heat source is *Q*_met_, and the external heat source is *Q*_ext_ (W/m^3^). In this model, constant metabolism and fat perfusion were taken into account, in order to model the *in vivo* experimentation. Dielectric and thermal tissue properties as well as the parameters used in the FEM model are described in Table 1 [23].

A linear solver was used to solve the FEM models carried out in this study. The mesh used to generate each model had 0.15 mm minimum element size, with 3410 elements and 26,449 degrees of freedom. 1.3 GHz Intel Core i5 4 GB 1600 MHz DDR3 RAM MacBook Air was used to process the model.

### 2.3. Phantom Preparation and Characterization

Breast phantom was prepared by dispersing 30 mL oil, 6 mL detergent, 10 mL tri-distilled water, and 0.3 g agarose. The mixture was heated up to 80°C to dissolve agarose; then, it was poured into a glass beaker. The phantom was solidified at room temperature [19]. In order to validate the phantom properties, its dielectric properties were measured and compared with those from the human breast tissue. The dielectric properties were measured by using the Hewlett Packard dielectric probe kit (85070C) connected to a network analyzer (Agilent Technologies, model E5071B). The permittivity sensor, which is inserted into the phantom, is able to measure real (*ε*) and imaginary (*ε*^”^) permittivity. Therefore, by using (4), the electrical conductivity was obtained.

(4) $$\begin{array}{c} \sigma =\varepsilon^{\prime \prime }\varepsilon_{0}\omega . \end{array}$$

### 2.4. Experimental System Used for Phantom and In Vivo Tissue Tests

The radiation system is the system that generates the microwave power applied to the tested tissue. The ISYS245 microwave generator and power amplifier (Emblation Microwave, Scotland, UK) were used to apply the microwave radiation at 2.45 GHz. Besides, this equipment monitored the transmission and reflection power levels. The standing wave ratio (SWR) was measured by using an Agilent Technologies network analyzer (E5071B).

The thermometry system is the system where real-time temperature was measured every second by using two Luxtron optic-fiber thermal probes (Luxtron STB MAR05, USA) during microwave ablation (MWA) experiments. Optic fiber thermal probes do not interfere with microwave radiation. The first thermal sensor was placed next to the antenna, between slots, while the second sensor was placed at 1.00 cm away from the first one along the antenna axis. The same configuration was used for every experimental test.

**Figure 2:**
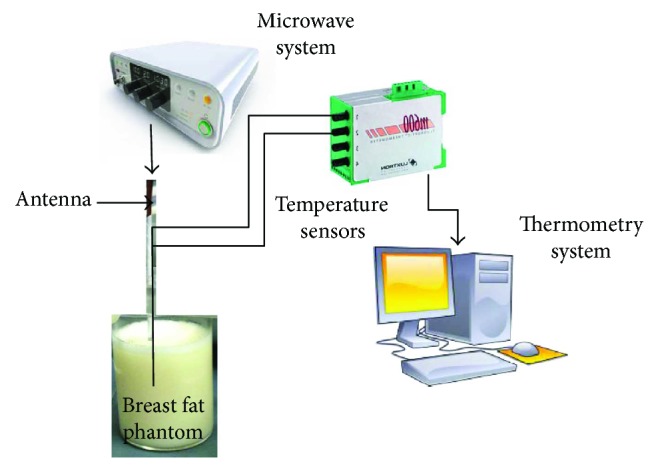
Phantom experimental setup. The radiation system provided the microwave power to generate the tissue ablation, while the thermometry system measured the temperature increase during each experiment.

Figure 2 describes the experimental setup used for the experiments carried out in phantom and *in vivo* tissues. The differences between experiments only consist in the media in which the antenna was inserted.

#### 2.4.1. Phantom Experimentation

The coaxial slot antenna and two optic fiber thermal probes were inserted inside the phantom. The antenna was fixed by using a homemade support. Four experiments were performed according to Table 2. The phantom experimentation setup is described in Figure 2, which describes the radiation and thermometry systems.

#### 2.4.2. In Vivo Breast Swine Experimentation

To assess the feasibility of using this antenna in clinical treatments, it is necessary to verify its performance in an *in vivo* model. The estimation of the effect of different input powers and exposition times is also important. A 90 kg, 4-year-old, multiparous potbellied Vietnamese minipig sow was used for the *in vivo* experimental test of this antenna. The sow was handled following the institutional guidelines; an ad hoc committee previously approved the experimental protocol. In order to induce deep anesthesia, the sow was treated with azaperone (IM) and tiletamine-zolazepam (IV). The coaxial slot antenna was inserted into the breast gland tissue by using an image-guided ultrasound system (Prosound 6, Hitachi Medical Systems, Switzerland). For each experiment, the antenna insertion was set when the antenna slots had been placed in the swine mammary gland tissue.  Table 2 describes the radiation parameters (power and time) and antenna insertion used in each experiment. It is important to mention that the antenna insertions were different because the amount of tissue varied depending on the location. The exposition time was determined by the temperature rise; the optic fiber sensors do not support temperatures higher than 100°C.

**Figure 3:**
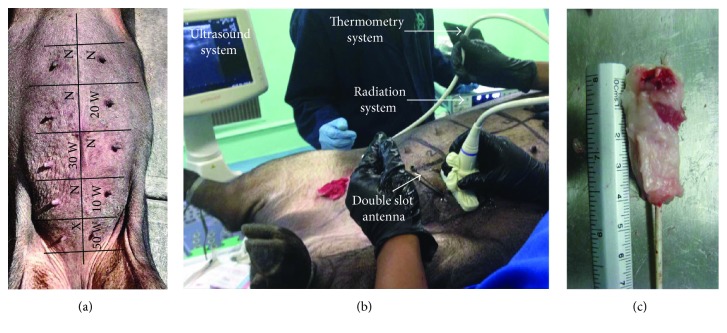
*In vivo* swine mammary gland experimentation. (a) Quadrants used to divide the sow mammary glands, depicting the arrangement of the experimental zones. Ventral quadrants were used for 10 W and 50 W, and thoracic quadrants for 20 W and 30 W. Alternate quadrants were used as controls without treatment (N). The first experiment with an error was performed in the X quadrant, and it was not analyzed. (b) The experimental setup consists of the radiation system and the thermometry system. The coaxial antenna was inserted by using an image-guided ultrasound system. (c) Example of the four regions analyzed to evaluate the thermal ablation effect. Region 1 (above) was the section where antenna slots were set, while region 4 (below) was the superficial section (3 cm from the antenna slots).

The sow mammary glands were exposed, disinfected, and divided into 10 quadrants (see Figure 3(a)), which included one nipple each. The microwave treatment was applied over each quadrant with different time and power conditions. 10 W and 50 W were applied at ventral quadrants, while 20 W and 30 W at the thoracic ones. Alternate quadrants were used as controls without treatment (N). The antenna was inserted through a small incision made in the skin, about 1 cm away of the nipple; the antenna was introduced with a 45° angle in a rear-lateral direction. The correct direction of the antenna was monitored by ultrasound imaging in order to identify the mammary tissue, which was localized about 2.5 cm below the skin. Once the antenna was placed, the radiation system was turned on at the selected radiation power. The thermometry system stored temperature data every second during the experiment time.  Figure 3(b) describes the *in vivo* breast swine experimentation. In order to analyze the effect of the ablation treatment over different tissue regions, the tissue around the antenna was segmented in four regions.  Figure 3(c) shows an example of a mammary gland obtained after the experimental procedure; here, it is possible to observe the four regions (about 1 cm long) that were established according to their distance from the position of the antenna slots. Region 1 (above) was the section where antenna slots were set, while region 4 (below) was the superficial section (3 cm from the antenna slots).

After the treatments, the animal was humanely euthanized. The mammary gland area was dissected separately by quadrants in the laboratory. To analyze the effect of the thermal ablation over the treated tissue, four different regions (1 cm), according to their distance from the insertion point, were studied. The first one was established as the deepest and the hottest, while region 4 was the most superficial and the coolest. A region without treatment was used as a control. Each sample was fixed in buffered formaldehyde for 24 hours at room temperature and embedded in paraffin for a histological study. The samples were cut in 5 *μ*m-thick slices, which were stained with haematoxylin and eosin for microscopic examination. At least three slides per region were obtained in order to evaluate the diameter of the damage and cell modifications in the tissue.

## 3. Results

### 3.1. Antenna

**Figure 4:**
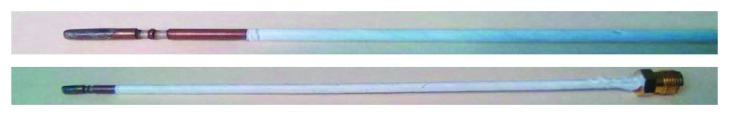
Double short distance slot antenna.

The election of the microwave antenna was based on the measurement of the standing wave ratio (SWR). We compare four different coaxial slot antennas: dipole, single-slot antenna, double-slot antenna, and double “short distance” slot antenna. The SWR measurements were carried out by inserting the antenna in breast tumor phantom surrounded by healthy breast fat.  Table 3 summarizes the SWR obtained for each antenna. The best SWR result was the one for the double short distance slot antenna (1.077).  Figure 4 depicts the tested double short distance slot antenna.

### 3.2. Phantom

**Figure 5:**
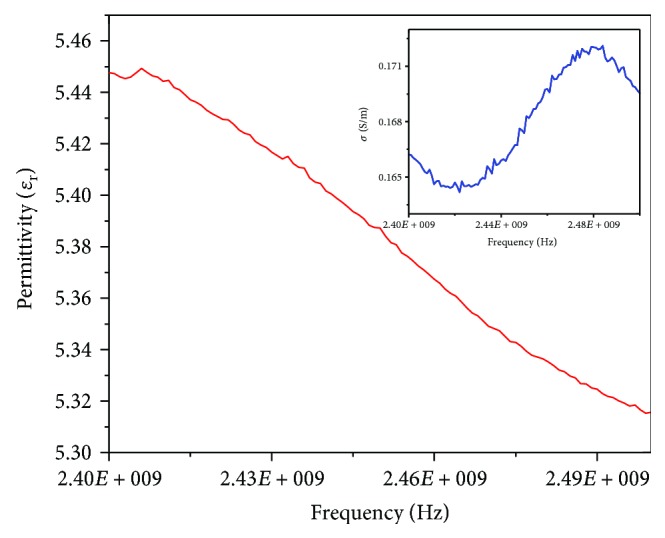
Relative permittivity and electrical conductivity of the breast tissue phantom measured from 2.40 GHz to 2.50 GHz.

Figure 5 describes the dielectric properties of the breast phantom implemented to evaluate the antenna behavior. Relative permittivity and electrical conductivity were measured from 2.4 GHz to 2.5 GHz. According to the literature, the properties of the breast tissue at 2.45 GHz are 5.14 and 0.14 S/m for relative permittivity and electrical conductivity, respectively. The relative permittivity of the phantom, at the same frequency, was 5.38, while the electrical conductivity was 0.16 S/m. It can be observed that the phantom is a good representation of the breast tissue dielectric properties.

### 3.3. FEM Thermal Distributions

**Figure 6:**
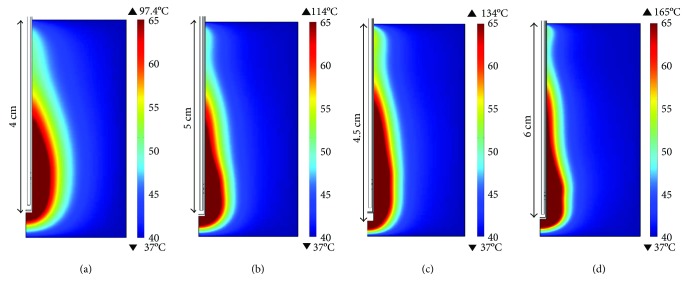
Temperature distributions in breast tissue generated by the 2D axisymmetric FEM model. (a) 10 W applied during 120 s at 4 cm of antenna insertion, (b) 20 W applied during 40 s at 5 cm of antenna insertion, (c) 30 W applied during 30 s at 4.5 cm of antenna insertion, and (d) 50 W applied during 15 s at 6 cm of antenna insertion.

Figure 6 shows temperature distribution in breast tissue obtained by the computational model, according to the scenarios described in Table 2. It is possible to observe that the thermal pattern depends on the input power and the exposition time.  Figure 6(a) shows the thermal distribution obtained for 10 W and 120 s; in this case, the heat was uniformly distributed. The heated area that reaches temperatures above 60°C corresponds to 0.89 cm^2^. For the cases with higher input power and lower exposition time, the heat distribution was concentrated over a thin region.  Figure 6(b) shows the thermal distribution for 20 W and 40 s; in this case, the area of the tissue over 60°C was 0.75 cm^2^. The bigger heated area (area = 0.96 cm^2^) was achieved with 30 W and 30 s. Finally, Figure 6(d) presents the thermal distribution obtained with an input power of 50 W applied for 15 s; the heated area was 0.80 cm^2^. It was observed that the antenna insertion and the treatment time are parameters that play important roles over the shape of the thermal distributions, while the level of input power is related with the reached temperatures.

### 3.4. Temperature Profiles

**Figure 7:**
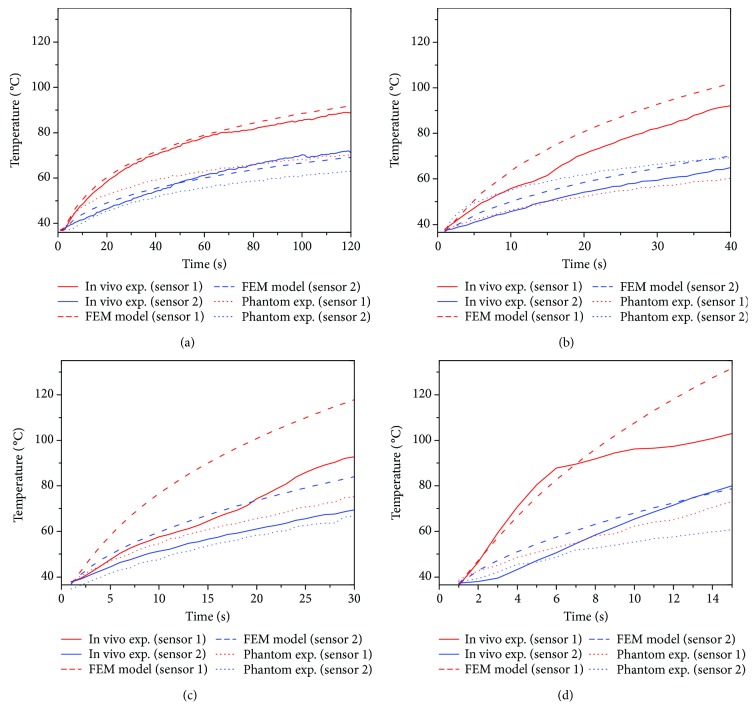
Comparison of the temperature profiles obtained for the experimentation *in vivo*, phantoms, and the FEM models. (a) 10 W applied during 120 s at 4 cm of antenna insertion, (b) 20 W applied during 40 s at 5 cm of antenna insertion, (c) 30 W applied during 30 s at 4.5 cm of antenna insertion, and (d) 50 W applied during 15 s at 6 cm of antenna insertion. Sensor 1 was located in the middle point between the slots, and sensor 2 at 1 cm of sensor 1, following the antenna axis.

Figures 7(a)–7(d) show a comparison between the temperature profiles measured during the phantom and *in vivo* experimentation and the FEM models. In all the scenarios, sensor 1 was located at the middle point between both slots, while sensor 2 was 1 cm above sensor 1 (following the antenna axis). The temperature was recorded every second during the experimentation time.  Figure 7(a) describes the temperature profiles for an antenna insertion of 4 cm and 10 W applied during 120 s. In this case, it is possible to observe a good agreement between *in vivo* experimentation and FEM modelling. Even for an antenna insertion of 5 cm and 20 W applied during 40 s, it is observed that the *in vivo* experiment and the FEM model tend to follow a similar behavior (see Figure 7(b)). However, if higher input power and lower experimentation times are evaluated, the difference between *in vivo* experiments and FEM models tends to increase, as observed in Figures 7(c) and 7(d). In the four cases, the differences between *in vivo* experiments, FEM models, and phantom experimentation were bigger; this can be due to the phantom that does not present perfusion and metabolism; that is, the phantom is a more ideal representation of the real tissue.

Table 4 shows the maximum temperatures recorded by the thermal sensors 1 and 2. As it was described, four different microwave powers and radiation times: 10 W (120 s), 20 W (40 s), 30 W (30 s), and 50 W (15 s), for both phantom and *in vivo* swine experimentation were carried out. Table 4 also presents the temperature difference for the FEM model versus phantom experimentation, the FEM model versus *in vivo* experimentation, and phantom versus *in vivo* experimentation. FEM models and *in vivo* experimentations were similar when low input power was applied for longer treatment times. The best case was when 10 W was applied per 120 s; the maximum differences were 2.74°C and 3.53°C for sensors 1 and 2, respectively. When the input power increases and the treatment time decreases, the difference starts to increase; however, sensor 2 presented lower temperature differences than sensor 1. FEM models for 30 and 50 W reached temperatures over 100°C, 109°C, and 136°C, respectively. However, the reached temperatures for the *in vivo* experimentations were 90.42°C and 103.96°C, respectively.

### 3.5. Micrographics

**Figure 8:**
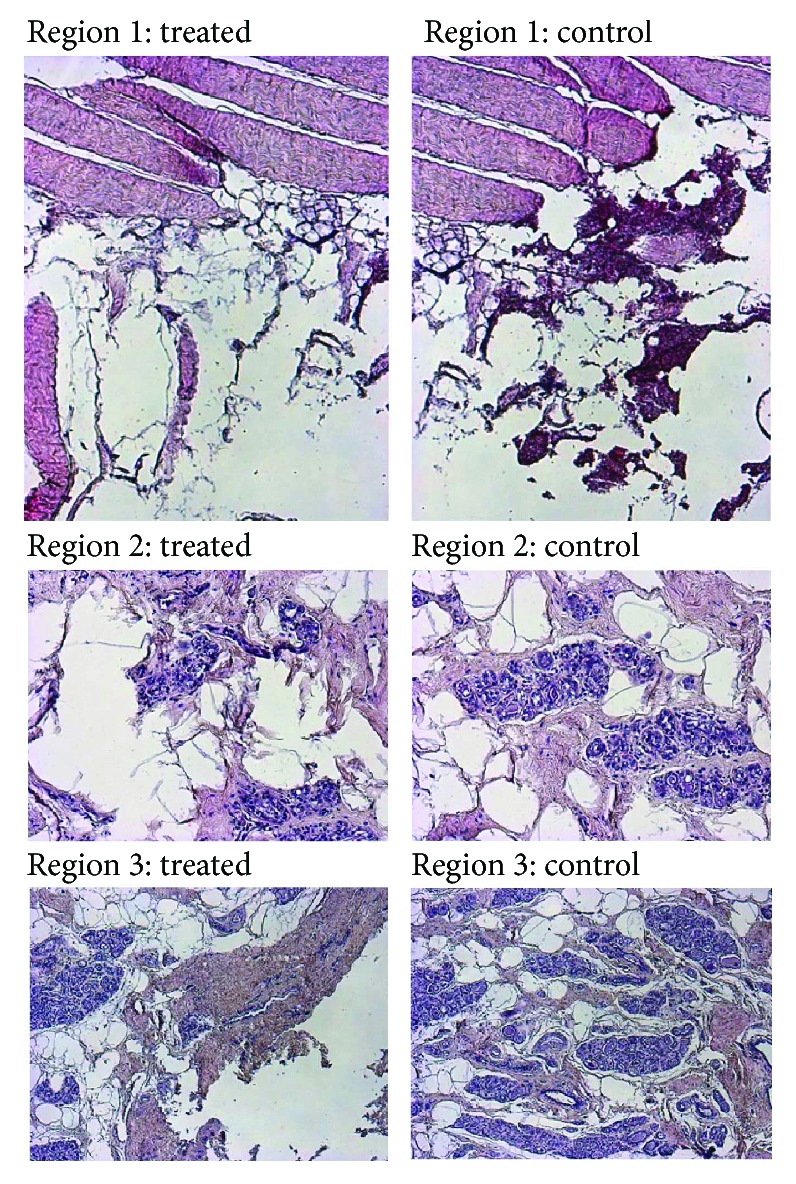
Microphotographs (100x) of sow control and treated (50 W) mammary gland samples showing regions 1, 2, and 3. Regions 2 and 3 were established at 1 cm and 2 cm from the antenna slots, respectively.

Figure 8 shows representative micrographs of the porcine mammary gland samples taken from regions 1, 2, and 3 from control and treated areas. From this analysis, it was observed that regions 1 and 2 are the most damaged ones. Region 1 was heavily damaged in muscular fibers and adipose tissue with hemorrhagic focal zones. For treated region 2, the connective tissue showed an orange coloration, which is not the coloration for normal tissue (pink). The difference in the coloration of the tissue was because of the ablation treatment. Region 3 also showed a slightly orange coloration, which means that this antenna is able to generate ablation over the tissue located at 2 cm from the antenna slots. In the control samples, tissue damage was not observed; neither the parenchyma nor the stroma presented changes.

According to the analysis based on the micrographics, the tissue damage of the four regions (in which the tissue was divided) was established.  Table 5 summarizes the results of the tissue damage score, considering the input power, the treatment time, and the antenna insertion. Region 1 represents the deepest antenna insertion zone, and region 4 the shallowest one. These results show that the highest ablation was obtained at the deepest zones (1 and 2), being the time of the application of the equalizer in the results.

## 4. Discussion and Conclusion

The application of microwave energy is recently developed in tumor ablation. Microwave ablation is a promising treatment for breast cancer because it produces local heat in the cancerous injury without damaging the surrounding healthy tissue. The overheating of the tissue can be due to the difference of the electric properties between healthy and tumor breast tissues. Microwave ablation is a treatment modality for liver, lung, kidney, and bone tumors; however, there are not many research reports regarding its application in breast cancer. In order to validate the double short distance slot coaxial antenna for breast cancer microwave ablation therapy, several tests were carried out. These tests allow us to study the feasibility of its use in the clinical practice. We present the computational model, phantom experimentation, and breast swine *in vivo* experimentation by using four different microwave powers (50 W, 30 W, 20 W, and 10 W) applied during different times (15 s, 30 s, 40 s, and 120 s, resp.). The finite element method (FEM) was used to perform a computational model. We modeled a double short distance coaxial slot antenna to find heat transfer in breast tissue under the same conditions applied during *in vivo* experimentation. Phantom experimentation was carried out in breast phantom, in order to evaluate the antenna performance under ideal conditions. Finally, the *in vivo* experimentation was carried out in a 90 kg swine sow.

The double-slot antenna described in this article was chosen because a previous study carried out by our laboratory showed that this antenna provided the best performance [22]. The distance between the antenna slots was adjusted to ensure a good coupling during treatment, no damage to the equipment, and to have the best energy transmission. The SWR of this antenna was 1.077 which was the best value in our study. The worst antenna (single slot) had an SWR of 2.98.

The computational model showed higher temperatures than expected. In fact, the higher input power values (30 W and 50 W) had the higher reached temperatures; just when low input powers (10 W and 20 W) were applied during longer times (120 s and 40 s), the reached temperatures were in the range for thermal ablation. It is important to remark that temperatures over 100°C can be reached because, although the models include tissue perfusion and metabolism, the thermal dependence of this was not taken into account. In order to improve the thermal simulations, a complete study of the thermal dependence not only of the thermal properties but also of the dielectric ones must be done. These are more complex models that use more computational resources and take more time. In the future, we will work on this model development which includes the thermal dependence of tissue thermal and dielectric properties.

The results obtained from the experimentation in the phantom gave us the certainty that the proposed antenna generated temperatures around the thermal ablation. However, due to the phantom as just an ideal representation of breast tissue, some differences were expected between these results and those from the *in vivo* experimentation. The phantom of breast tissue was based on the electric properties, and the thermal properties were not considered for its preparation. It is necessary to find components to mix into the phantom in order to resemble the real tissue thermal properties. Therefore, in order to test the antenna behavior in a model closer to the reality, we conducted an *in vivo* experiment. A histological analysis that would allow us to verify the tissue damage, according to microwave power and exposure time, was carried out. It is important to emphasize that during the *in vivo* experimentation the reflected power in all cases was lower than 1 W. This value ensures a maximum power transmission to the breast tissue.

As it was expected, higher levels of power generated tissue ablation in shorter times. The tissue damage, according to the histological analysis, was higher near the slots, and it decreases as a function of distance. These results are consistent with those obtained from the FEM model. In all cases, we observed a similar pear shape, but for higher powers (30 W and 50 W), there was a heating in the proximal region of the antenna; that is, the pear shape was more elongated. According to the model, there is a direct relationship among power, time, and temperatures: it is possible to obtain higher temperatures in shorter times by using a higher power; in this case, the temperature at the proximal region of the applicator kills the cells around that area. In the *in vivo* experiment, the times to get ablation temperatures next to the slots were approximately 20 s for 10 W, 15 s for 20 W, 12 s for 30 W, and 2 s for 50 W. According to these results, it could be advisable to use lower power in order to only ablate the cancerous area without damaging the surrounding healthy tissue.

The experimental evaluation (*in vivo* and phantom) and the FEM model results suggest that MWA using this novel optimized applicator could be successfully used to eliminate precise and small areas of cancerous tissue (around 20–30 mm^2^). The ablation procedure could be directed with high precision in order to eliminate substantial parts of a tumor and reduce the damage of the surrounding healthy tissue. By modulating the power and treatment time, it might be possible to increase/decrease the ablation area. It is important to address the importance of the improvement of FEM models in order to use it as a tool for treatment planning. Finally, we conclude that, despite the differences obtained in experimental and computational results, it is feasible to use this optimized applicator in breast cancer treatment when applying the correct power and time to get the best heating pattern to ablate only tumor cells. These results suggest that clinical experimentation might be started.

## Figures and Tables

**Table 1:** Parameters used in the development of the computational model.

Parameter	Value
Breast electric conductivity	0.14 S/m
Breast relative permittivity	5.14
Breast thermal conductivity	0.42 W/mK
Breast density	1020.00 kg/m^3^
Breast metabolic heat rate	3.9 W/m^3^
Blood density	1000 kg/m^3^
Blood specific heat	3639 J/(kg·K)
Breast perfusion rate	33 mL/min/kg
Blood temperature	37°C
Dielectric relative permittivity	2.03
Catheter relative permittivity	2.60
Microwave frequency	2.45 GHz
Radiation time	4 min
Input microwave power	Variables (10, 20, 30, and 50 W)

**Table 2:** Radiation parameters and antenna insertion used in each experiment (phantom and *in vivo* tissue experiments).

Experiment	Radiation power [W]	Radiation time [sec]	Antenna insertion [cm]
1	10	120	4
2	20	40	5
3	30	30	4.5
4	50	15	6

**Table 3:** Coaxial slot antenna SWR measurements.

Slot antenna	SWR [W]
Dipole	2.117
Single slot	2.986
Double slot	1.847
Double short distance slot	1.077

**Table 4:** Maximum temperature in the thermal sensor next to the antenna slots for the computational model, phantom experimentation, and *in vivo* swine experimentation.

	FEM model	Phantom experimentation	*In vivo* experimentation	FEM model versus phantom	FEM model versus *in vivo*	Phantom versus *in vivo*
Power/time (W/s)	Maximum temperature next to the slots (°C)			ΔT (°C)		
10/120	92.11	70.91	89.37	21.20	2.74	18.46
20/40	102.60	69.15	91.31	33.45	11.29	22.16
30/30	109.20	76.78	90.42	32.42	18.78	13.64
50/15	136.00	75.22	103.96	60.78	32.04	28.74

Power/time (W/s)	Maximum temperature at 1 cm (°C)			ΔT (°C)		
10/120	69.35	63.60	72.88	5.75	3.53	9.28
20/40	70.40	59.84	64.61	10.56	5.79	4.77
30/30	84.85	67.11	69.45	17.74	15.40	2.34
50/15	80.60	63.71	85.27	16.89	4.67	21.56

**Table 5:** Tissue damage score in samples of sow mammary gland undertaking thermal ablation.

Power-time	10 W-120 s		20 W-40 s		30 W-30 s		50 W-15 s	
Group	Ctl	Treated	Ctl	Treated	Ctl	Treated	Ctl	Treated
Region								
1	Neg	+++	Neg	++++	Neg	++++	Neg	++++
2	Neg	++	Neg	++	Neg	++	Neg	++++
3	Neg	+	Neg	++	Neg	+	Neg	++
4	Neg	+	Neg	+	Neg	+	Neg	+

++++: heavy damage; +++: defined damage; ++: moderate damage; +: low damage; Neg: no damage; Ctl: control.

## Data Availability

The data used to support the findings of this study are available from the corresponding author upon request.
